# Turkish validity and reliability study of the nursing home adjustment scale

**DOI:** 10.1186/s12877-023-04314-1

**Published:** 2023-09-29

**Authors:** Ceren Varer Akpinar, Tahir Belice, Selman Bolukbasi, Aliye Mandiracioglu

**Affiliations:** 1 Department of Public Health, Faculty of Medicine, Giresun University, Giresun, Turkey; 2 Department of Internal Diseases, University of Health Sciences İzmir Bozyaka Education and Training Hospital, İzmir, Turkey; 3 Department of Eldely Services, Family and Social Services Provincial Directorate, Manisa, Turkey; 4 Department of Public Health, Faculty of Medicine, Ege University, İzmir, Turkey

**Keywords:** Nursing homes, Adjustment, Factor analysis, Turkey

## Abstract

**Background:**

Adapting to a nursing home has a significant effect on facilitating the transition to a nursing home. However, a tailored nursing home adjustment scale for Turkish nursing home residents is not yet available. The purpose of this study was to translate the nursing home adjustment scale from English to Turkish and assess its reliability and validity in a sample of nursing home residents in Turkey.

**Methods:**

A total of 202 older adults from four nursing homes were included in this study. The reliability of the Turkish nursing home adjustment scale was tested using Cronbach’s alpha values for internal consistency. Exploratory and confirmatory factor analyses were used to examine the factor structure, whereas correlation analysis was conducted for convergent validity.

**Results:**

The Turkish version displayed satisfying internal consistency (Cronbach’s alpha = 0.88) and perfect convergent validity for life satisfaction (*r* = 0.482). The Turkish scale included five factors: difficulty fitting in, acceptance of new residence, emotional distress, depressed mood, and relationship development.

**Conclusion:**

The findings indicate that the Turkish version of the scale is a valid and reliable tool for measuring the level of nursing home adjustment among older adults in Turkey.

## Background

The demand for long-term institutional care is growing these days. Older adults begin to lose the ability to fend for themselves because of chronic illness and disability. Family care for older adults is declining as the economy improves and family structures change. Transition to nursing home life is considered as a stressful life event for the older adult [1–4]. Living in a nursing home is defined as ‘being away from all other experiences that older adult have accumulated over a lifetime’ [5].

The public structure of nursing home life and the restrictions imposed to comply with rules are among the most important factors complicating this process. This is because a person’s living environment changes dramatically, and there is an institution that has control over their life [6]. For an older adult, adapting to a new living environment requires learning new routines, participating in the institution’s activities, forming and maintaining new social relationships, and managing personal property [7].

Residents who adjust to nursing homes feel at home and express a sense of well-being; however, nursing home residents who are unable to adapt experience feelings of depression and are likely to display angry and aggressive behaviours [8]. Many transitional factors have been identified, including personal and environmental factors that affect nursing home adjustment [9, 10].

Adaptation is a developmental process that includes behaviours and sensations that arise in reaction to problems faced by older adult in a new place. Therefore, older adults interact with various transition factors in the dynamic process of adaption [5, 7]. Factors related to nursing home adjustment are one of the most crucial processes by which nursing home workers can provide active interventions [8]. Therefore, a direct assessment of nursing home adjustment using an convenient scale to determine problems is important in developing effective interventions to help older adults transition to nursing homes. Adjustment to nursing homes has historically been measured using a life satisfaction scale, which is generally an indicator of adjustment, rather than using an instrument specifically designed for this purpose. It has been stated in the past literature that factors affecting adjustment to nursing homes were examined by using the life satisfaction scale as an adjustment index. However, to assume that life satisfaction is an indicator of adjustment means ignoring factors of nursing home adjustment that are not reflected in life satisfaction. Direct assessment of adjustment to nursing home life by using the nursing home adjustment scale will allow the identification of factors independently associated with adjustment [4, 11]. The Nursing Home Adjustment Scale (NHAS) was developed in Korean language by Lee in 2007 [11]. It was later adapted into English and according to the cultural characteristics of different regions of China [7, 12, 13]. This scale is a psychometric tool for the quantitative measurement of the elderly's nursing home adjustment. It was used in studies among the elderly living in nursing homes as one of the most widely used measurement tools in this field.

Traditions, social customs, values, cultural norms and expectations vary for many cultural groups. For this reason, psychometric evaluations should be carried out before its use in different countries. There is no scale developed on this subject in Turkish. It is important to investigate the convenience of the scale developed by Lee to Turkish culture and language. The proportion of the elderly population in the total population is increasing rapidly in Turkey. It increased to 9.9% in 2022 and according to population projections, it is predicted to reach 12.9% in 2030 [14]. In addition, the need for institutional care for healthy elderly people is increasing in the country. It is stated that while the number of public nursing homes was 63 in 2022, this number increased 2.5 times and reached 158 in 2020 and their capacity increased 2.4 times [15].

This study aimed to translate the Nursing Home Adjustment Scale into Turkish and assess its reliability and validity in a sample of nursing home residents.

## Materials and methods

### Study participants

This study was conducted in four nursing homes in Manisa, Turkey. Nursing homes participating in the study are public. In Turkey, the standards of public nursing homes have been determined by a regulation [16]. Public nursing homes are divided into four groups according to their socioeconomic level. All four nursing homes in the study are in the districts of Manisa, have the same characteristics and serve the socioeconomically vulnarable population.

The inclusion criteria were: (1) ability to speak and (2) cognitively able to respond to the questionnaires and (3) healthy enough to carry on a conversation. Older adults with cognitive problems or major psychiatric disorders and poor hearing were excluded from the study. The population of study consisted of 205 people aged over 60 years living in four nursing homes in Manisa. The purposive sampling method was used in this research and the researchers aimed to reach all people aged over 60 years living in four nursing homes. The response rate of the study was 98.5% (202 older adult).

### Data collection and instrument

Data were collected through face-to-face interviews with older adults between May and August 2020. The interviews were held in a private room, and each interview lasted approximately 20 minutes. The researcher who conducted the interviews worked actively in one of the nursing homes and was experienced. During the interview, each statement of the questionnaire was read aloud by researcher. The participants were asked to respond verbally, and the answers were then objectively written. The data-collection form consisted of three parts. The first part included questions prepared by the researchers about the sociodemographic characteristics of the participants, their disease status, medical drug use, and health perception. Self-rated health was assessed using a single item, ‘In general, how would you rate your health?’ [17].

In the second part, the ‘Satisfaction with Life Scale’ was used, which was previously studied for Turkish validity and safety by Dağlı et al. [18]. The scale consisted of five 7-point Likert-type questions. As the total score increased, life satisfaction increased. The Cronbach’s alpha coefficient of the one-dimensional scale was 0.88.

The third part of the questionnaire included the Nursing Home Adjustment Scale (NHAS). The original version of the adjustment for nursing home residents was initially published in Korean [11], subsequently the English version was generated [7]. The Cronbach’s alpha value of the 5-point Likert-type English NHAS consisting of 23 propositions was 0.77. The items were grouped into five dimensions: emotional distress, difficulty in fitting in, relationship development, acceptance of new residences, and depressed mood. Some items were scored inversely, and the higher the total score, the better the fit.

In this study, the Turkish NHAS was developed in two main stages. The first stage consisted of scale translation and back-translation. The second stage was used to evaluate the instrument’s psychometric properties.

### Scale translation

In the cross-cultural adaptation of the NHAS for Turkish older adults, firstly, an independent advanced translation from English to Turkish was carried out by a translator who was fluent in both Turkish and English. Subsequently, retrospective translation by two translators whose native language was English provided a consistent translation. Finally, the committee, consisting of a linguist, internist, public health specialist, and social worker, reviewed all versions and agreed on the translation. The acceptability of the final version was confirmed by individuals living in an institution other than a nursing home, which was taken as a sample, using a pilot application.

### Statistical analyses

The Cronbach’s alpha coefficient and item-total scale correlations were evaluated for internal consistency in the Turkish reliability evaluation. Item-total correlations assume that an item should have a correlation coefficient of at least 0.2 with the domain it belongs to. A Cronbach’s alpha value of ≥0.70 is considered evidence of scale internal consistency, and higher values indicate greater reliability [19].

Convergent validity, known group comparisons, and factor structure were investigated for validity. Exploratory factor analysis (EFA) and confirmatory factor analysis (CFA) were used to examine the factor structure of the Turkish NHAS with the Turkish nursing home population.

Kaiser-Meyer-Olkin (KMO) tests were used to control the suitability for factor analysis, and Bartlett tests were used to evaluate the probability of high correlation between scale items. KMO values exceeding 0.5 indicate sufficient sample sizes for factor analysis [20]. For EFA, by applying varimax rotation according to the principal components method, items with factor loads >0.40 within the factor structures were evaluated. To control double loading, a difference between the factor loads of at least 0.10 was accepted. While naming the factors that emerged as a result of EFA, the factors names in the original scale and the factors on which the items accumulated were considered, and the items were named according to the meaning they expressed. Some fit indices and standardized regression coefficients were examined by performing CFA to test whether there was an adequate relationship between the factors determined by the EFA, which variable was associated with which factor, whether the factors were independent of each other, and whether the factors were sufficient to explain the model. For model fit, the ratio of chi-square to degrees of freedom (χ2/df), root mean square error of approximation (RMSEA), and comparative fit index (CFI) were calculated. The cut-off values of good fit for these indices were < 0.08 for RMSEA, > 0.90 for CFI, and < 2.0 for the ratio of chi-square to degrees of freedom (χ2/df). Since chi-square statistics are affected by sample size, χ2/df ratio was preferred.

Correlation analysis was used to examine the convergent validity of the NHAS with life satisfaction. Correlation values ≥ 0.45 were considered acceptable for convergent validity with a similar construct [7, 19].

In the known group comparison validity, the distinguishability of the scale was demonstrated by evaluating the relationship between independent variables, such as sociodemographic characteristics and the average length of stay in a nursing home, and the scores of the NHAS. Normal distribution of data was evaluated with the Shapiro-Wilk and Kolmogorov-Smirnov tests. Because the data were not normally distributed, Mann-Whitney U and Kruskal-Wallis tests were used to evaluate relationships between the scores of the NHAS and independent variables. Dunn's Test was used as a Post-hoc Test for the variables with significant Kruskal-Wallis test results. In pairwise comparisons, the level of significance was accepted as *p*<0.016 with Bonferroni correction. SPSS Amos 24.0 was used for confirmatory factor analysis and SPSS 25.0 was used for other analyses. The analysis results were evaluated within the 95% confidence interval, and the statistical significance limit was accepted as *p* < 0.05.

### Ethics

The study was conducted in accordance with the guidelines and regulations of the Helsinki Declaration for Ethical Principles of Research. This study was approved by the Medical Research Ethics Committee of Ege University (April 3, 2020, no: 20-4T/5) and written informed consent was obtained from all participants. In addition, official permission was obtained from the institutions affiliated which the nursing homes are affiliated. Permission to work on the Turkish version of the scale was obtained from the author who developed the English scale.

## Results

The descriptive characteristics of the participants are presented in Table 1. Of older adult, 69.8% were male, predominantly between the ages of 75–84, and were primary school graduates (Table 1).

**Table 1:** Characteristics of the participants (*n* = 202)

	n (%)	Mean ± SD
**Age**		77.89 ± 7.78
60–64	5 (2.5)	
65–74	64 (31.7)	
75–84	87 (43.1)	
≥85	46 (22.8)	
**Gender**		
Female	61 (30.2)	
Male	141 (69.8)	
**Educational Status**		
Illiterate	47 (23.3)	
Literate	38 (18.8)	
Primary education	97 (48.0)	
High school	8 (4.0)	
University and above	12 (5.9)	
**Economic Status**		
Bad	70 (34.7)	
Moderate	67 (33.2)	
Good	65 (32.2)	
**Place of stay**		
Rural	65 (32.2)	
Urban	137 (67.8)	
**Marital Status**		
Single	44 (21.8)	
Divorced	48 (23.8)	
Widow	98 (48.5)	
Married	12 (5.9)	
**Length of stay (months)**		44.69 ± 50.19
<12	70 (34.7)	
12–36	60 (29.7)	
>36	72 (35.6)	
**Toilet fulfilment**		
Independent	192 (95.0)	
Dependent	10 (5.0)	
**Nocturia**		2.60 ± 1.64
**Number of outpatient visits in the last year**		5.31 ± 3.46
**Number of hospitalisations in the last year**		0.71 ± 1.50
**Life Satisfaction Scale**		26.22 ± 5.37
**Health Perception**		
Very Bad	2 (1.0)	
Bad	14 (6.9)	
Moderate	77 (38.1)	
Good	90 (44.6)	
Very Good	19 (9.4)	

*SD* Standard Deviation

The Cronbach’s alpha value for the entire scale was 0.88. The Cronbach’s alpha values of factors 1, 2, 3, 4, and 5 were 0.87, 0.84, 0.84, 0.83, and 0.79, respectively. When each item was deleted, Cronbach’s alpha values similarly changed between 0.87 and 0.88. The deletion of any item could not improve its reliability; therefore, no items were deleted. The item-total scale correlations range from 0.26 to 0.71 (Table 2).

**Table 2:** Item distribution characteristics, exploratory factor structure, and internal consistency data of the Turkish NHAS

	**Mean (SD)**	**Factor Loadings**					**Alpha if the item was deleted**	**Item-Total Correlation**
		**F1**	**F2**	**F3**	**F4**	**F5**		
**Factor 1. Difficulty fitting in (Cronbach’s alpha = 0.87)**								
13. It is difficult to get along with the other residents	2.32 (1.33)	**0.93**	0.11	0.13	0.02	0.10	0.87	0.65
4. I don't like attending group activities	2.31 (1.30)	**0.92**	0.12	0.12	0.08	0.11	0.87	0.64
14. I don't want to live here	1.57 (1.19)	**0.81**	-0.04	0.16	-0.01	-0.04	0.88	0.47
23. I think about my previous home and it makes me sad	2.74 (1.54)	**0.75**	0.10	0.24	0.25	0.05	0.87	0.71
5. This is not my home, but only a temporary accommodation	1.90 (1.39)	**0.66**	0.08	0.14	0.21	0.12	0.87	048
12. Daily living has no meaning for me	2.18 (1.49)	**0.53**	0.12	0.20	0.17	0.04	0.87	0.50
**Factor 2. Acceptance of new residence (Cronbach’s alpha = 0.84)**								
17. I have accepted living here	1.27 (0.50)	0.04	**0.91**	0.08	0.10	0.01	0.88	0.35
16. I want to live well here	1.37 (0.56)	0.06	**0.89**	-0.01	0.02	0.18	0.88	0.32
11. I'm glad to be here	1.24 (0.42)	0.02	**0.88**	0.07	0.06	0.18	0.87	0.36
7. I am at ease here	1.43 (0.58)	0.14	**0.84**	0.10	0.11	0.13	0.10	0.44
15. My life has meaning in the world	1.56 (0.69)	0.17	**0.66**	0.15	-0.01	-0.12	0.88	0.30
**Factor 3. Emotional distress (Cronbach’s alpha = 0.83)**								
2. I usually feel angry	2.35 (1.29)	0.31	0.16	**0.84**	0.12	0.08	0.87	0.67
6. I am not friendly with the other residents	2.37 (1.28)	0.21	0.05	**0.79**	0.13	0.11	0.88	0.54
9. I am bored with living here	1.94 (1.37)	0.30	0.12	**0.72**	0.12	0.08	0.87	0.61
3. I get upset for little things	2.39 (1.45)	0.13	0.17	**0.71**	0.25	0.25	0.87	0.61
**Factor 4. Depressed mood (Cronbach’s alpha = 0.84)**								
21. I usually feel like crying	2.55 (1.49)	0.03	0.06	0.13	**0.87**	-0.11	0.87	0.39
22. I shed tears without reason	2.35 (1.55)	0.11	0.05	0.20	**0.84**	-0.11	0.87	0.45
20. It is painful for me to think about my children and their families	2.73 (1.54)	0.08	0.05	0.09	**0.81**	0.08	0.88	0.42
19. I usually feel lonely	2.35 (1.37)	0.24	0.07	0.06	**0.68**	0.10	0.87	0.47
**Factor 5. Relationship development (Cronbach’s alpha = 0.79)**								
18. I have a sincere friend here	1.96 (1.19)	0.02	0.07	0.07	0.03	**0.84**	0.88	0.29
10. I try to help people who live here	2.05 (0.84)	0.11	0.22	-0.17	0.05	**0.77**	0.88	0.26
8. I want to make friends here	1.78 (0.83)	0.05	0.04	0.27	-0.12	**0.74**	0.88	0.28
1. I am friendly with my friends in the nursing home	2.19 (1.26)	0.14	0.03	0.40	0.09	**0.70**	0.88	0.41
**Cronbach’s alpha = 0.88**								

A priori target loading designed to measure each factor are in bold

Bartlett tests < 0.001

Kaiser-Meyer-Olkin (KMO) coefficient: 0.782

The KMO value calculated before the EFA was 0.78 and Bartlett’s test of sphericity was significant (*p* < 0.001). In the EFA, applied to indicate the construct validity of the Turkish version of the scale, the factor loadings of all 23 items in the scale were found to be higher than 0.40. In the Turkish version, one cross-loading item (Item 1) on two factors was found. However, this item was not removed from the scale because the difference between the loads they assigned to the factors was greater than 0.10. The EFA revealed that the five-factor structure explained 68.17% of the total variance.

**Fig. 1:**
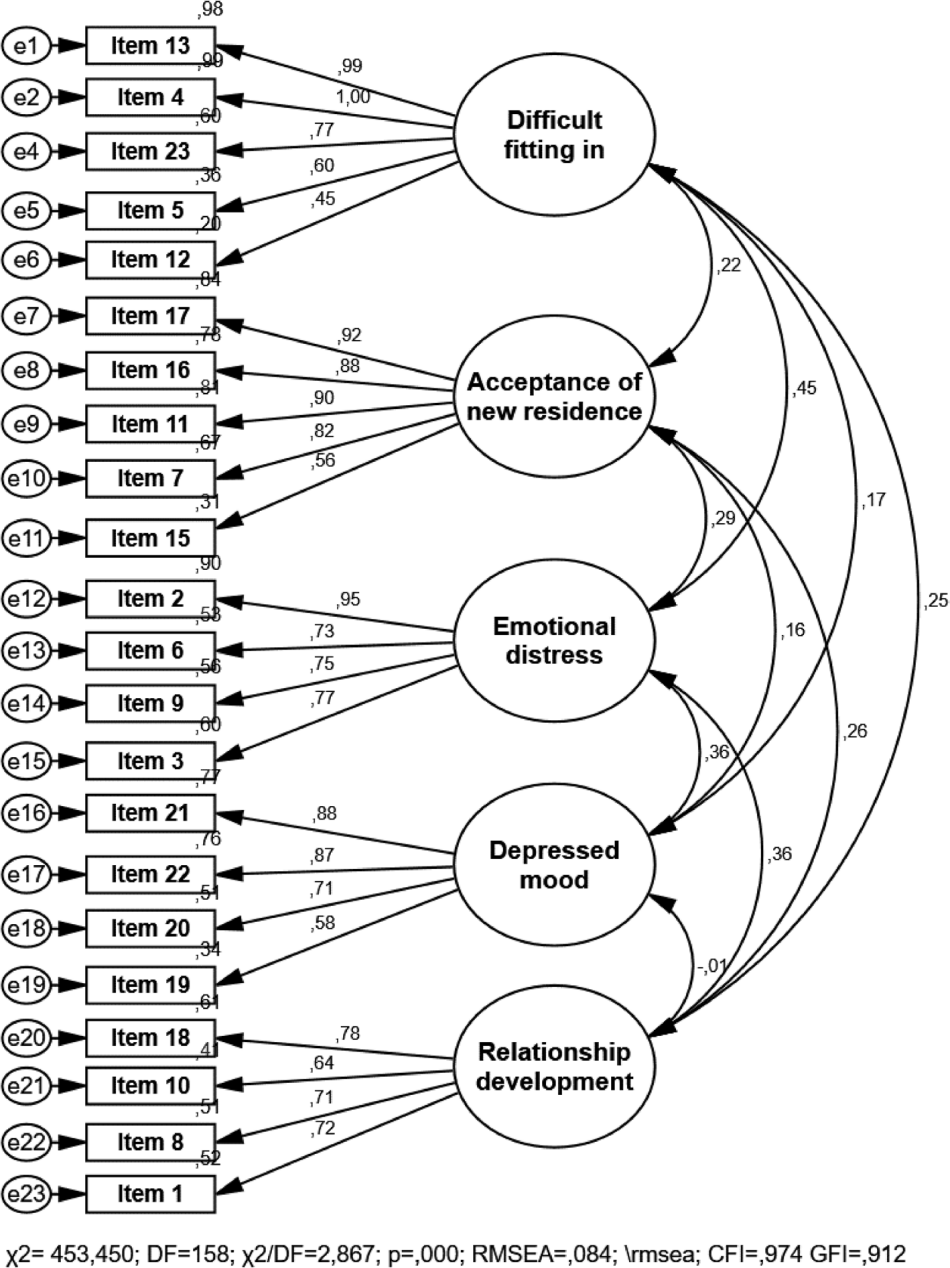
Confirmatory factor analysis For structural equation modelling solutions with five factors, all parameter estimates are standardised

After the EFA, a CFA was performed. As a result of the CFA, the approximate fit indices for the five-factor model were not within the desired limits of the approximate fit indices (*χ2 = 687,403*; *df = 220; χ2/df = 3,125; p = ,000*; *RMSEA = ,103; CFI = ,875*; *GFI = ,816*), therefore, the modification indices were examined. Consequently, Item 14 under Factor 1 was removed from the scale, as it had a covariance relationship with the other factors and items below them. All the model fit indices obtained in the subsequent repeated analysis met the acceptability criteria (*χ2 = 453,450*, *df = 158, χ2/df = 2,867, p = ,000*, *RMSEA = ,084; CFI = ,974*; *GFI = ,912*) (Fig. 1). And the final structure for the 22 items was grouped into five domains entitled difficulty fitting in, acceptance of new residence, emotional distress, depressed mood, and relationship development.

The correlation between the NHAS and satisfaction with life scale scores was evaluated for convergent validity. The NHAS and its sub dimensions had a statistically significant correlation with the satisfaction with life scale scores (*respectively r* = 0.482, *r* = 0.573, *r* = 0.467, *r* = 0.414, *r* = 0.392, *r* = 0.492, *p* < 0.001), indicating that the NHAS had substantial levels of convergent validity with life satisfaction.

The "known groups" validity findings of the scale are presented in Table 3. In the final version of the scale with 22 questions, the highest score that can be obtained from the scale was 110, and the lowest score was 22. The mean total NHAS score of the participants was 69.09 ± 11.91 (minimum: 46, largest: 103). Significantly higher scores on the NHAS were found for those living in the city, for those with a high education level, for those who stated their economic status as moderate/good, and for those who stayed in a nursing home for less than 12 months. (*p* < 0.05) (Table 3).

**Table 3:** Known group comparison results of the Turkish NHAS

	**Median (25-75 percentile)**	**p**
**Age**		0.181*
60–74	78.00 (65.00-87.00)	
75–84	77.00 (65.00-81.00)	
≥85	71.50 (61.00-80.00)	
**Gender**		0.405**
Female	78.00 (65.50-83.50)	
Male	76.00 (62.00-85.00)	
**Educational Status**		0.001*
Illiterate	68.00 (58.00-78.00)^a^	
Primary education	72.00 (68.00-75.00)	
High school and above	78.00 (66.00-85.00)	
**Economic Status**		0.040*
Bad	73.00 (61.50-80.00)^a^	
Moderate	76.00 (62.00-81.00)	
Good	78.00 (67.00-87.00)	
**Living place**		0.001**
Rural	65.00 (59.00-75.50)	
Urban	79.00 (69.00-86.50)	
**Marital Status**		0.102**
Single-Divorced-Widow	76.50 (62.00-85.00)	
Married	79.50 (70.00-87.00)	
**Length of stay (months)**		0.001*
<12	80.50 (68.00-87.00)^a^	
12–36	69.00 (61.00-79.00)	
>36	70.00 (63.75-80.75)	
**Health Perception**		0.210*
Very Bad- Bad	67.50 (56.50-84.50)	
Moderate	76.00 (62.00-85.00)	
Good-Very Good	78.00 (66.00-85.00)	

^*^ Kruskal Wallis

^**^Mann Whitney U

^a^The group with significant difference as a result of Post Hoc Analysis (Dunn Test)

In addition, the scores of the NHAS sub-dimensions were found to be significantly higher in those with a high level of education, those who stated their economic situation as good, and those who stayed in a nursing home for less than 12 months (*p* < 0.05).

## Discussion

This study is the first in Turkey to develop and evaluate the Turkish version of the NHAS. It has been understood that the Turkish version has acceptable reliability and validity to directly measure the adjustment of individuals living in nursing homes in Turkey. The Turkish NHAS displayed satisfactory internal consistency (Cronbach’s α = 0.88). While the reliability of the Turkish version NHAS was higher than the English (Cronbach’s α = 0.77) and Korean (Cronbach’s α = 0.83) version, it was similar to the Chinese (Cronbach’s α = 0.87) version 1 [7, 11, 12]. In the Chinese version 2 study, the Cronbach alpha value was found to be higher, as 0.91 [13].

The NHAS validated in this study consisted of five factors, and was similar to the English version [7]. Only after CFA was one item not included in the scale because it showed covariance with items in other factors and thus the scale consisted of 22 items. After a CFA, the final scale model was found to be compatible. The subscales were in good agreement with the previous theoretical and conceptual framework. In addition, Cronbach’s alpha coefficient for each of the five factors of the scale was sufficient (> 0.70). Therefore, the general NHAS and its subscales could be used in Turkish. Besides that, adaptation studies of NHAS in different cultures and languages should be conducted to compare the factor structures.

The 5 factors (“difficulty fitting in nursing home”, “acceptance of new residence”, “emotional distress”, “depressed mood”, “relationship development”) that emerged in this scale demonstrate the nursing home adjustment process very well. Because adjustment to a nursing home is not only a positive or negative mood; it is stated that it brings with it a more complex psychological process [21]. The period of adjustment following a move to a nursing home is seen by many as a process that leads to the loss of independence, autonomy, decision-making and the continuity of old roles. The loss of an individual's home life poses a great challenge that threatens his identity, belonging and sense of self [22]. In a qualitative study, it is stated that the elderly want to connect with others in their new environment and at the same time reconnect with the family. It is emphasized that adapting to the rules and regulations of the nursing home and trying to develop new social networks is an additional source of stress and anxiety for some people [22].

The scores of the elderly from the sub-dimensions of this scale can also provide clues about how the elderly should be prepared for the nursing home process by the staff.

The average life satisfaction scale score of the elderly people included in the study was calculated as 26.22 ± 5.37. In a study conducted in another city in Turkey using the same scale, the life satisfaction score of the elderly living in a nursing home was determined as 14.4±4.81 [23].This study demonstrated the significant convergent validity of the Turkish NHAS with the Life Satisfaction Scale. This means that residents who are well-adjusted to the nursing home are likely to have high life satisfaction. It was used for the convergent validation of the NHAS in the English validity and reliability study [11]. Other studies have also revealed that elderly people with higher life satisfaction have better nursing home adjustment [24, 25].

According to the analysis carried out for the known group validity, the scale was able to distinguish between the adjustment of older adult with different characteristics to the nursing home. Based on this, it was determined that those who were better educated, those who stated their economic status as moderate and good, and those who stayed in a nursing home for less than one year had significantly higher scores on the NHAS. This situation reflects the cultural characteristics of our country: those with good socioeconomic status and education prepare themselves for institutional care in advance; those with low socioeconomic status have more expectations from their families, and it is natural that they have challenges adapting to nursing homes. On the other hand, in a study conducted in China, it was reported that the elderly who stayed in a nursing home for more than six months had higher adjustment scores [25]. According to the results of the systematic review examining the factors affecting the adjustment to the nursing home, the compliance of the more educated elderly and those with a longer stay is better. In this review, they explain this situation as follows: *“probably, nursing home residents gradually reorganize and adjust to their nursing home life after an initial period of overwhelmed, disorganized, and emotionally reactive”* [21].

This study had several limitations. A generalisation cannot be made due to the use of purposive sampling in the selection of the participants. Furthermore, because older adult with cognitive problems were excluded, their compliance was not evaluated. Therefore, it is necessary to develop different methods to understand the adaptation of this special group to nursing homes. As in similar studies, the responses may have been affected by factors such as social desirability, as the collected data were based on the statements of the participants.

Despite these limitations, this study supports the psychometric properties of this useful Turkish tool to measure nursing home adjustment among residents in Turkey. An increasing number of older adult are receiving institutional care, and assessing their compliance is important to the well-being of life in the institution. Identifying nursing home adjustment and associated factors helps ensure effective interventions and better adjustment of older adults living in nursing homes.

## Conclusion

It has been established that the Turkish version of the NHAS has acceptable reliability and validity to evaluate the adjustment of individuals living in nursing homes in Turkey.

## Data Availability

The datasets used and/or analysed during the current study are available from the corresponding author on reasonable request.
